# Early bloom, early boom? Motor competence profiles across maturation: a cross-sectional study

**DOI:** 10.5114/biolsport.2026.160855

**Published:** 2026-07-06

**Authors:** Matteo Giuriato, Alessandro Gatti, Agnese Pirazzi, Caterina Cavallo, Vittoria Carnevale Pellino, Marco Del Bianco, Roberto Codella, Nicola Lovecchio, Matteo Vandoni

**Affiliations:** 1 Laboratory of Adapted Motor Activity (LAMA), Department of Public Health, Experimental Medicine and Forensic Science, University of Pavia, 27100 Pavia, Italy; 2 Department of Education and Sport Sciences, Pegaso University, 80143 Naples, Italy; 3 National PhD Programme in One Health approaches to infectious diseases and life science research, Department of Public Health, Experimental and Forensic Medicine, University of Pavia, Pavia, 27100, Italy; 4 Department of Sport, LUNEX University of Applied Sciences, Avenue du Parc des Sports, Differdange, 4671, Luxembourg; 5 Department of Biomedical Sciences for Health, Università Degli Studi di Milano, 20133 Milan, Italy; 6 Department of Endocrinology, Nutrition and Metabolic Diseases, IRCCS MultiMedica, 20138 Milano, Italy; 7 Department of Human and Social Science, University of Bergamo, 24127 Bergamo, Italy

**Keywords:** Motor coordination, Growth and development, Movement proficiency, Physical education, School-aged children

## Abstract

Motor competence (MC) evolves throughout childhood as biological maturation progresses, yet previous research has largely approached this relationship linearly. Whether more complex patterns exist remains unclear, and while higher body mass index (BMI) is associated with lower MC, its contribution to maturityrelated differences is undetermined. This study examined the relationship between biological maturation and basic MC (BMC) and explored whether BMI mediates this association in schoolchildren. A total of 1155 Italian children (665 girls; age = 9.8 ± 2.0 years) completed anthropometric measurements and the MOBAK assessment battery during physical education classes. Biological maturation was estimated using maturity offset equations and expressed as years from peak height velocity (PHV). Quadratic regression models were used to test the association between maturation and BMC. Mediation analysis was conducted to determine whether BMI z-score mediated the relationship between maturation and BMC. Quadratic modeling demonstrated that maturation was non-linearly associated with BMC (β = 0.279, p < 0.001), indicating a U-shaped trajectory, with lower competence observed near PHV and higher values pre- and post-PHV. BMI z-score was negatively associated with BMC (β = -0.196 to -0.220, p < 0.001) but did not mediate the maturation-BMC relationship. Sex differences emerged by domain, with girls outperforming boys in self-movement tasks and boys scoring higher in object-movement tasks. Maturation shows a non-linear association with BMC, characterized by a temporary decline around PHV. Although BMI is negatively related to BMC, it does not explain maturation effects. These findings highlight PHV as a sensitive developmental period during which targeted motor skill support may be needed in educational and sport settings.

## INTRODUCTION

It is widely acknowledged that children exhibit diverse morphological types and growth trajectories, making the evaluation of biological rather than chronological age an essential consideration in pediatric research [1]. These growth processes do not progress uniformly, as developmental changes occur in non-linear and individual-specific ways [2]. Biological maturation also plays a crucial role during childhood, influencing both children’s physical development and physical capacities [3, 4]. Given these effects, consideration of maturation is essential in applied settings, allowing practitioners to align training loads and routines with each child’s unique developmental pace [5]. Among the commonly used indirect indicators of maturation, the maturity offset and Peak Height Velocity (PHV) provide an easy and cost-effective means to estimate biological age [6]. Combining this information, Lloyd et al. recommend PHV-based training plans corresponding to the phases before, around, and after PHV to promote balanced physical growth [1,7].

These maturational changes are not only reflected in physical growth but also in the development of motor competence (MC), which also evolves throughout development [8]. Since maturation influences neuromuscular control, strength, and coordination, it plays a central role in shaping MC trajectories [9]. MC refers to a broad latent concept reflecting the degree of proficiency in motor skills and their underlying mechanisms, all essential for active engagement in sports and physical activities [9]. The relevance of MC can be summarized by a theory from Stodden and colleagues [10]. Their theory concludes that poor MC in children lead to a negative spiral of disengagement, which leads to reduced physical activity and physical fitness, leading to a higher risk of obesity and the development of chronic diseases [10]. To confirm this, different studies have linked a higher level of MC during childhood to improved physical fitness and greater adherence to physical activity [11]. Moreover, others have observed the reverse relationship, finding that lower MC is associated with reduced activity levels and an increased risk of obesity [12, 13]. To gain a better understanding of the developmental foundations of MC, researchers often examine basic motor competencies (BMC), which are fundamental movements that form the basis of children’s participation in sport and physical education. [14]. These competencies are typically evaluated through basic tasks such as throwing, catching, balancing, and running, which represent the fundamental requirements for organized movement activities and foster physical literacy [15]. BMC can thus be viewed as a focused expression of MC, specifically designed for educational settings, emphasizing the essential MC required for successful participation in both school-related and extracurricular physical activities [16–18]. The progression of BMC is closely interconnected with biological maturation, as maturational changes in body size, proportions, strength, and neuromuscular control directly affect how children acquire and refine motor skills [19, 20]. However, the relationship between biological maturation and BMC remains a subject of ongoing debate, and evidence suggests it may not be strictly linear across developmental stages [21]. Notably, previous studies have relied on linear analytical approaches [22, 23], which may oversimplify maturation-related processes that are inherently non-linear, particularly around PHV. Such models may fail to capture phase-specific accelerations and decelerations in development, potentially contributing to inconsistent findings in the literature [24]. Moreover, excess adiposity is associated with lower BMC and may attenuate the positive effects of biological maturation [21, 24]. Despite these interconnected influences, the combined effects of biological age, Body Mass Index (BMI) z-score, and BMC have rarely been explored simultaneously. Understanding these relationships across early and middle childhood is essential to design effective interventions and informing educational practice. To address this gap, the present study, analyzed cross-sectional data collected from Italian schools to explore (1) the relationship between maturity (measured in years from PHV) and BMC, with specific attention to its potential non-linear nature, and (2) whether BMI and maturation act as mediators in this complex relationship with MC.

## MATERIALS AND METHODS

### Study design

This study is based on cross-sectional data from a longitudinal study started in 2023 [25] and conducted in a Northern Italian region to evaluate the BMC and weight status of kindergarten, primary and middle school children. All the study procedures were approved by the Lombardy Regional Ethics Committee 6 (Prot. N. 0025557/25) and adhered to both the Strengthening the Reporting of Observational Studies in Epidemiology (STROBE) guidelines (Table S1) [26], and the Declaration of Helsinki [27].

### Participants

Between November 2023 and April 2025, schoolchildren (5–13 years) from different educational institutions in North Italy were recruited for participation. After a thorough explanation of the study’s procedures, verbal assent was obtained from the children, alongside written informed consent from their parents or legal guardians. All evaluations were conducted during scheduled Physical Education (PE) classes. Assessments were administered by postgraduate students in Sport Science, each of whom received detailed training and instructions to ensure standardized procedures. Children were included in the study if: i) regularly attended school and PE classes, ii) had no contraindications or injuries that might restrict their ability to participate in physical activities and iii) understood the study procedures and instructions. Exclusion criteria encompassed any orthopedic injury within the previous six months, medical conditions contraindicating physical activity, or any limitation preventing participation in PE. No academic credits were offered for participation, and students could withdraw of the study at any point without any negative consequences.

### Procedures

A total of 1155 children (no missing data for all the outcomes) were assessed, including 665 girls (57.6 %), ranging from preschool to middle school. All assessments took place within the sports facilities of the participating schools and included both anthropometric measurements and BMC evaluation. Participants were instructed to arrive on the day of testing having eaten only a light, traditional Italian breakfast. Before the start of assessments, a standardized overview of the study procedures was delivered to all children involved.

### Anthropometric characteristics

Anthropometric assessments were conducted in accordance with the standards established by the International Society for the Advancement of Kinanthropometry (ISAK) [28]. Participants were evaluated while barefoot and dressed in lightweight exercise clothing (shorts and T-shirt). Body mass was measured using a calibrated digital scale (Seca 864, Seca GmbH & Co., Hamburg, Germany) with a precision of 0.1 kg. Height was recorded to the nearest centimeter using a stadiometer (Seca 216, Seca GmbH & Co., Hamburg, Germany), with participants standing in an upright position and the head aligned according to the Frankfort horizontal plane. The collected measurements were subsequently used to compute the body mass index (BMI) z-scores, according to the World Health Organization (WHO) Growth Standards.

### Maturity offset

To estimate maturity offset, we applied Moore’s formula:

$$\begin{array}{c} \text{For girls}:\\ -7.709133+(0.0042232\times (\text{Age}\times \text{Height})) \end{array}$$

$$\begin{array}{c} \text{For boys}:\\ \text{Maturity Offset}=-7.999994+(0.0036124\times (\text{Age}\times \text{Height}) \end{array}$$.

Following this, the predicted age at PHV was calculated by subtracting the maturity offset from each participant’s chronological age, as outlined by Kozieł and Malina and used in previous studies [6,29]. Based on years from PHV, participants were classified as pre-PHV (early maturers, -3.50 to -0.51 years), on-time (-0.50 to +0.49 years), or post-PHV (late maturers, +0.50 to +3.49 years).

### MOBAK test

The MOBAK assessment tool measures BMC using eight distinct tasks, which are divided into two core domains: object manipulation and self-movement skills [14]. Each domain consists of four tasks that assess specific aspects of BMC. The object control domain includes throwing, catching, bouncing, and dribbling, while the selfmovement domain comprises balancing, rolling, jumping, and running [30]. The protocol features age-appropriate assessments tailored for different educational stages: MOBAK KG for preschoolers, MOBAK 1–2 for 1^st^ and 2^nd^ class, MOBAK 3–4 for 3^rd^ and 4^th^ class, and MOBAK 5–6 for students in 5^th^ class and 6^th^ class. Each task is scored from 0 to 2 points, with a maximum of 8 points per domain and a total possible score of 16 points, reflecting overall MC. A detailed description of the MOBAK test has been provided elsewhere [14] and in Table S3.

### Statistical analysis

Descriptive characteristics were summarized as means and standard deviations (SD), while categorical variables were reported as frequencies and percentages. To identify the most appropriate functional form for the association between biological maturation and BMC, three alternative models were compared: a linear regression, a quadratic regression, and a generalized additive model (GAM) with penalized splines. Model fit was evaluated using the Akaike Information Criterion (AIC). The quadratic model yielded the lowest AIC value (AIC = 6156.95), followed by the GAM (AIC = 6158.43) and the linear model (AIC = 6177.06), indicating that the quadratic specification provided the best overall fit. Based on these results, the association between biological maturation and BMC was evaluated using quadratic regression models, with maturation status considered as years from PHV. Both linear and quadratic terms were included to account for potential curvilinear relationships. Regression models were conducted separately for total BMC, object-movement, and self-movement subdomains. Two models were examined: Model 1 included BMI z-score as a covariate, while Model 2 further adjusted for sex (reference: girls). Unstandardized coefficients (b), standardized coefficients (β), 95% confidence intervals (CI), and p-values were reported for all predictors. The significance threshold was set at p < 0.05. To further examine potential indirect pathways, a mediation analysis was conducted using a path model to assess whether BMI z-score mediated the relationship between maturation, sex, and MC. Standardized path coefficients and p-values were estimated, with statistical significance set to p < 0.05. Significant pathways were represented as solid lines and non-significant paths as dashed lines in the path diagram. To complement the continuous analyses based on years from PHV, maturation groups differences in total BMC, object-movement and self-movement scores were examined using analysis of covariance (ANCOVA), with maturation group as the fixed factor and sex included as a covariate. Assumptions of normality and homoscedasticity were evaluated using Shapiro-Wilk and Breusch-Pagan tests. When violations were detected, heteroskedasticity-consistent (HC3) robust standard errors were applied. Posthoc pairwise comparisons were conducted using estimated marginal means with Holm correction for multiple testing. Adjusted p-values < 0.05 were considered statistically significant. Group-wise results are presented graphically in Figure S1. All statistical analyses were performed using R software (version 4.4.0; R Foundation for Statistical Computing). Data visualization and diagnostic plots were generated using the “mgcv”, “ggplot2”, “lavaan”, “emmeans” and “DiagrammeR” packages.

## RESULTS

The descriptive characteristics of the total sample, divided by sex, is reported in Table 1. The table also presents the sex distribution across maturation categories (pre-PHV, around PHV, and post-PHV), showing that most of both girls and boys were classified as pre-PHV (86%), with a higher proportion of late maturers observed among girls compared with boys.

**Table 1:** Descriptive characteristics of the total sample, grouped by sex.

	Girls (n = 665)	Boys (n = 490)	All the sample (n = 1155)
**Mean (95% CI)**			

Age (years)	9.4 (9.3, 9.5)	10.3 (10.2, 10.4)	9.8 (9.7, 9.9)
Weight (kg)	34.4 (33.6, 35.2)	38.5 (37.5, 39.4)	36.1 (35.5, 36.7)
Height (cm)	138.6 (137.7, 139.5)	144.0 (143.0, 145.0)	140.9 (140.2, 141.6)
BMI z-score	0.32 (0.23, 0.41)	0.52 (0.41, 0.64)	0.41 (0.34, 0.48)
Maturity offset (years)	−2.13 (-2.24, -2.01)	−2.59 (-2.70, -2.48)	−2.32 (-2.41, -2.24)

**Maturity status**			
Pre-PHV	551 (82.9%)	446 (91.0%)	997 (86.3%)
On time	46 (6.9%)	32 (6.5%)	78 (6.8%)
Post-PHV	68 (10.2%)	12 (2.4%)	80 (6.9%)
Object movement score	4.0 (3.8, 4.1)	4.9 (4.7, 5.1)	4.4 (4.2, 4.5)
Self-movement score	4.2 (4.0, 4.4)	3.2 (3.0, 3.3)	3.7 (3.6, 3.9)
Total MOBAK score	8.2 (7.9, 8.4)	8.039 (7.7, 8.3)	8.1 (7.9, 8.3)

Note: All data are presented as mean (95% confidence interval; CI). Kg, kilograms; cm, centimeters; BMI, body mass index.

Table 2 presents the results of the quadratic regression analyses examining the associations between maturation status (years from PHV) and BMC, including total BMC, self-movement, and object-movement subdomains. Across both models, the quadratic term for maturation was significantly and positively associated with all MC outcomes (*p* < 0.01), while the linear term was non-significant in all cases (*p* > 0.05), indicating a curvilinear (inverted U-shaped) relationship. Specifically, the strongest quadratic association was observed for total MC (Model 2: β = 0.279, *p* < 0.001), followed by self-movement (Model 2: β = 0.266, *p* < 0.001), object-movement (Model 2: β = 0.201, *p* = 0.001). BMI z-score showed a consistently negative and significant association with all MC outcomes in both models (*p* < 0.001), suggesting that higher BMI was related to lower MC. In Model 2, which included sex as an additional covariate, sex was a significant predictor for both self-movement (β = -0.263, *p* < 0.001) and object-movement (β = 0.200, *p* < 0.001), with boys performing better in object-movement tasks and girls outperforming boys in self-movement tasks. Sex was not a significant predictor of total MC (*p* = 0.222). These associations have been graphically represented to improve clarity (Figure 1 and 2).

**TABLE 2:** Quadratic regression analysis between basic motor competencies (object-, self-movement and the sum of the two) with maturation status (peak height velocity) in children of primary and middle school.

		Model 1			Model 2		

		b (95% CI)	β	P	b (95% CI)	β	P
Basic motor competencies	Maturation – Linear (years)	0.039 (-0.259, 0.338)	0.016	0.796	0.022 (-0.278, 0.322)	0.009	0.885
	Maturation – Quadratic (years)	0.202 (0.116, 0.288)	0.281	**< 0.001**	0.2 (0.114, 0.287)	0.279	**< 0.001**
	BMI z-score	-0.585 (-0.747, -0.424)	-0.199	**< 0.001**	-0.578 (-0.74, -0.416)	-0.196	**< 0.001**
	Sex (boys)	//	//	//	-0.251 (-0.654, 0.152)	-0.035	0.222

Self-movement	Maturation – Linear (years)	0.048 (-0.129, 0.225)	0.033	0.595	-0.03 (-0.201, 0.141)	-0.020	0.731
	Maturation – Quadratic (years)	0.119 (0.068, 0.17)	0.279	**< 0.001**	0.113 (0.064, 0.162)	0.266	**< 0.001**
	BMI z-score	-0.383 (-0.479, -0.288)	-0.220	**< 0.001**	-0.35 (-0.442, -0.258)	-0.200	**< 0.001**
	Sex (boys)	//	//	//	-1.131 (-1.361, -0.902)	-0.263	**< 0.001**

Object-movement	Maturation – Linear (years)	-0.008 (-0.195, 0.178)	-0.006	0.929	0.052 (-0.131, 0.235)	0.035	0.623
	Maturation – Quadratic (years)	0.083 (0.029, 0.137)	0.191	**0.003**	0.087 (0.035, 0.14)	0.201	**0.001**
	BMI z-score	-0.202 (-0.303, -0.101)	-0.113	**< 0.001**	-0.228 (-0.327, -0.129)	-0.128	**< 0.001**
	Sex (boys)	//	//	//	0.88 (0.634, 1.127)	0.200	**< 0.001**

Model 1 was adjusted only by BMI z-score. Model 2 was adjusted for BMI z-score and sex (reference girls). b = beta unstandardized coefficients. β = beta standardized coefficients. CI, confidence interval; BMI, Body Mass Index.

**FIG. 1:**
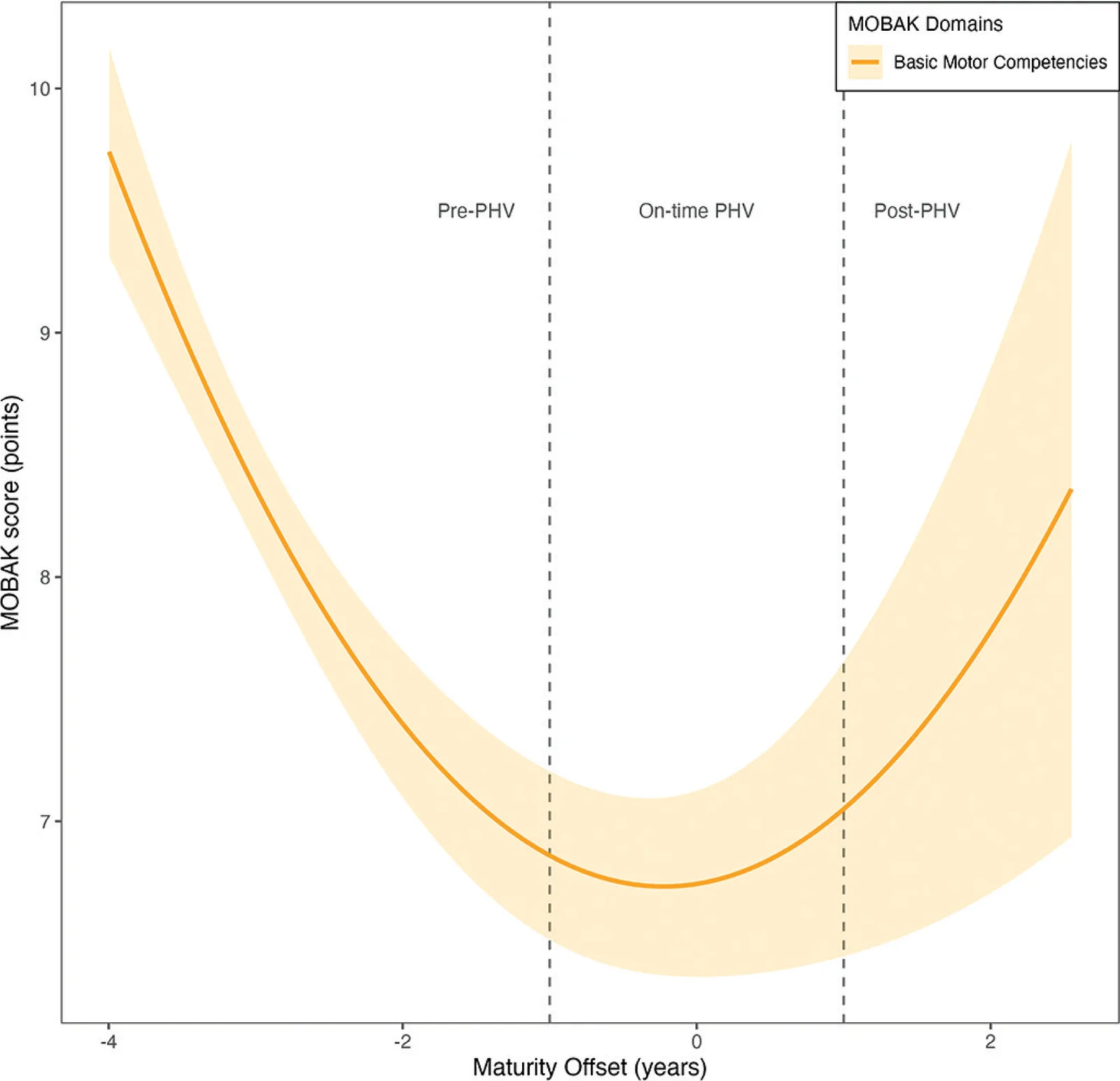
Association between Basic Motor Competence and maturity offset in children. Shading indicates the 95% confidence intervals of the association.

**FIG. 2:**
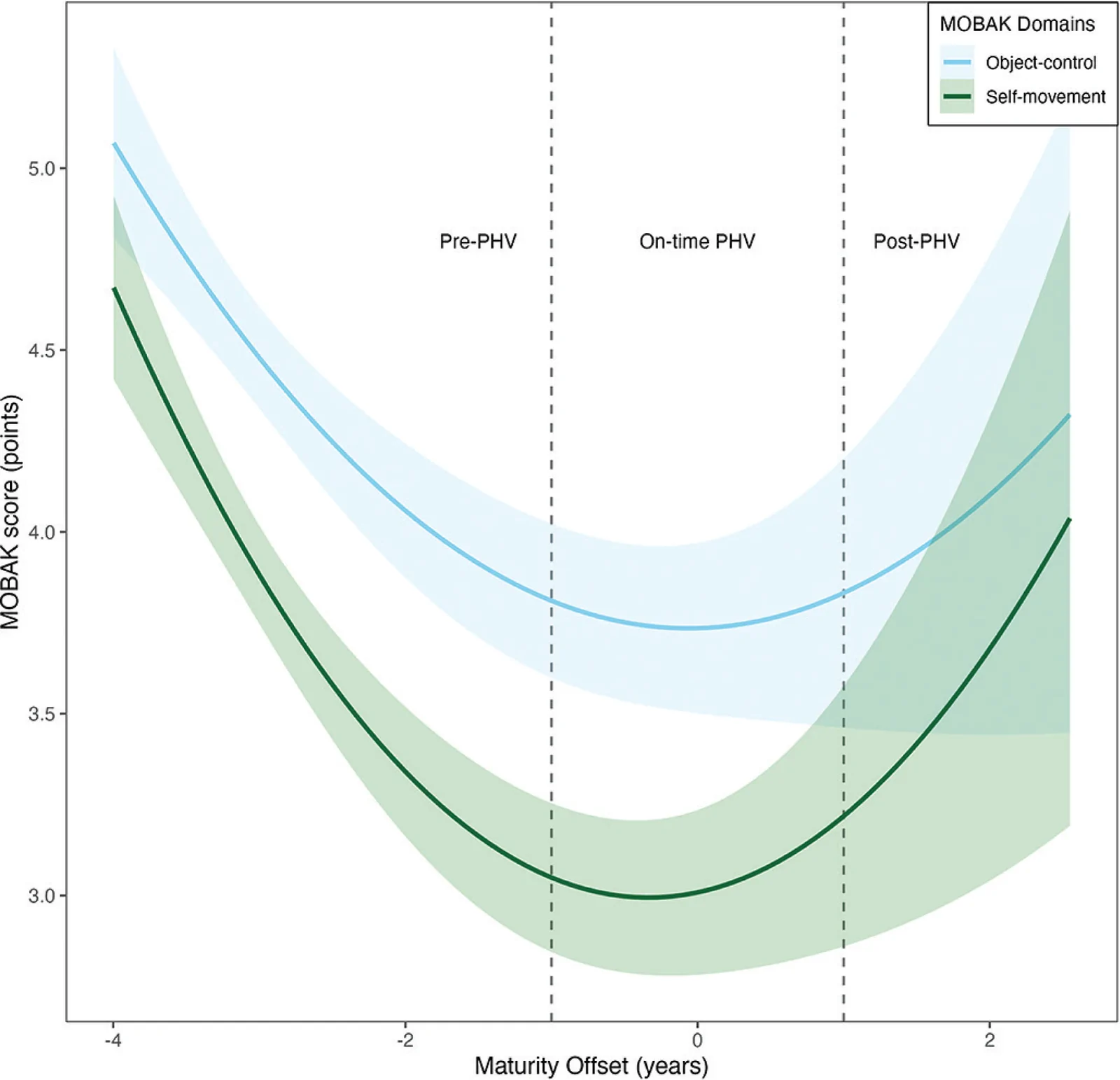
Association between object- and self-movement and maturity offset in children. Shading indicates the 95% confidence intervals of the association.

Figure 3 displays the results of the mediation analysis evaluating the direct and indirect effects of biological maturation (measured through years from PHV offset) and sex on MC, with BMI z-score included as a potential mediator. To identify the most appropriate structural model, we compared a full mediation model (excluding the direct pathway from maturation to motor competence) with a partial mediation model in which both direct and indirect effects were specified. The chi-square comparison indicated that removing the direct pathway significantly reduced model fit (Δχ^2^(1) = 69.18, p < .001), supporting retention of the partial mediation structure. The final model was saturated (df = 0), meaning that traditional global fit indices cannot be interpreted because saturated models reproduce the observed covariance matrix exactly [31]. Therefore, interpretation is based on the nested model comparison and the statistical significance of individual pathways. Maturation had a significant direct negative association with MC (β = -0.24, p < .001), while it was not indirectly related to MC through BMI z-score. No significant relationship between maturation and BMI z-score was found, however, BMI z-score itself had a significant negative association with MC (β = -0.20, p < 0.001), indicating that higher BMI was related to lower MC. Sex was significantly associated with BMI zscore (β = 0.08, p < 0.05), with boys showing higher BMI z-scores than girls, but had no significant direct effect on MC, nor an indirect effect through BMI z-scores. Overall, the mediation model highlights that BMI z-score does not mediate the relationship between maturation and MC. Sex, while influencing BMI, does not have a significant impact on MC in this model. Table S2 presents the parameter estimates from the mediation model, including direct, indirect, and total effects with bootstrapped confidence intervals.

**FIG. 3:**
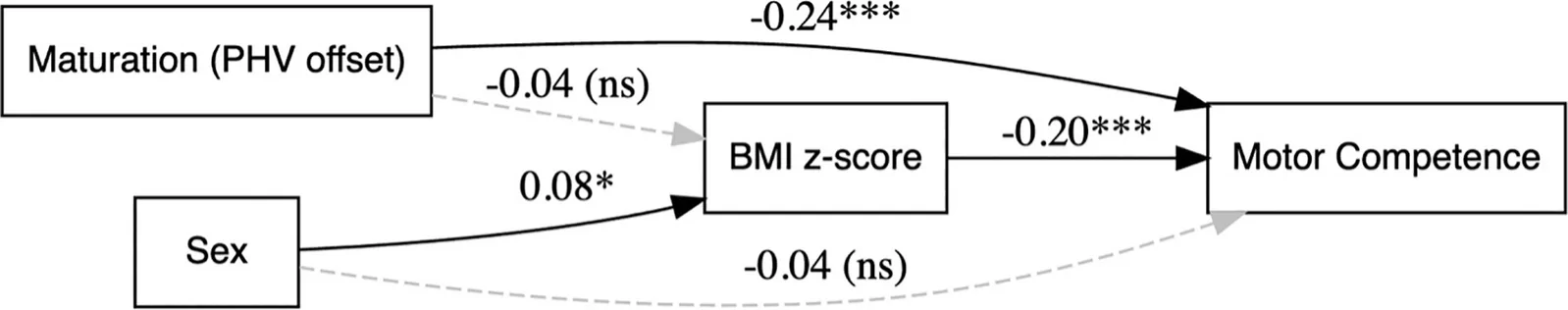
Path diagram of the mediation analysis examining the indirect and direct effects of biological maturation (PHV offset) and sex on motor competence, with BMI z-score as a mediating variable. Significant pathways are indicated by solid lines (p < .05; p < .001), and non-significant paths are shown with dashed lines.

Figure S1 illustrates adjusted group differences in BMC outcomes across pre-PHV (early maturers), on-time and post-PHV (late maturers). For object-movement skills, early maturers scored significantly higher than on-time maturers (mean difference = 0.68, p = 0.014), whereas no significant differences were observed between early and late (p = 0.318) or between late and on-time maturers (p = 0.318).

For self-movement skills, early maturers outperformed both late (mean difference = 0.79, p = 0.001) and on-time maturers (mean difference = 0.85, p = 0.001), while no difference emerged between late and on-time groups (p = 0.847). Similarly, total BMC scores were higher in early maturers compared with both late (mean difference = 1.10, p = 0.007) and on-time maturers (mean difference = 1.53, p < 0.001.

## DISCUSSION

The aim of this study was to explore potentially how biological maturation is related to BMC in early and middle childhood. Our results showed that a quadratic, U-shaped relationship better explained the relationship between BMC and maturation: BMC were higher in pre- and post-PHV children, but lower in their “on-time” peers. This pattern suggests that BMC do not follow a linear trajectory with biological age during the PHV period. A secondary aim of this study was to examine whether ponderal status could act as a mediator in the relationship between maturation and BMC. Although both maturation and BMI z-score were significantly related to BMC, the mediation analysis indicated that BMI did not significantly mediate the association between maturation and BMC. This suggests that while higher BMI is generally associated with lower MC, the maturational effects on MC occur largely independently of BMI, pointing toward other biological or neuromuscular mechanisms as primary drivers of this non-linear pattern.

Analyzing the association between different outcomes using nonlinear models is increasingly recognized as crucial in life-science research [32]. Linear approaches often fail to capture the variability and adaptive fluctuations characteristic of human development. In line with this perspective and with our results, recently Bandeira et al. proposed [33] an alternative view of MC as a more complex model to the traditional linear approach. The authors propose a multilayer framework, which clearly shows that MC should be understood as an emerging property of a complex system, in which biological factors (maturation, body composition), behavioral factors (activity levels, motivation), cognitive factors (perception of competence, self-efficacy), and environmental factors (practice opportunities, socio-cultural context) interact in a dynamic and non-linear way [33]. Within this framework, our findings emphasize the dynamic instability around the PHV period, likely reflecting temporary disruptions in coordination and neuromuscular control due to rapid growth. In line with our results, Sheehan & Lienhard [22] reported a decline in MC immediately after PHV for both boys and girls. Conversely, considering a multilevel linear model, Dos Santos et al. [23] reported an increase in gross MC with increased maturation. These differences from our results, aside from the use of a linear model, might be attributable to the methodology used to assess MC. While we used a test that allows the assessment of BMC, the KöperkoordinationsTest für Kinder used by Dos Santos et al. [23] focuses more on the evaluation of coordination rather than simple basic movements. Therefore, the divergence between our findings and those of Dos Santos et al. [23] may differ not only from model specification (linear vs non-linear) but also from the specific construct of MC measured. Whereas coordination tests emphasize complex, learned movement patterns, BMC assessments reflect underlying control and body management abilities. In addition, when considering our results for the two MOBAK domains separately, the non-linear pattern appeared more pronounced for selfmovement than for object-control skills. Specifically, self-movement scores exhibited a steeper U-shaped trajectory across maturation, suggesting that these abilities are more sensitive to maturational transitions, specifically around the PHV period. However, the higher BMC observed in pre- and post-PHV children should not be interpreted as a true developmental advantage of one group over the other, but rather as the result of a temporary reduction in motor competence around the PHV period. This interpretation is further supported by the categorical analyses, which showed that early maturers consistently displayed higher BMC scores than their on-time peers across all outcomes, whereas differences between late and ontime maturers were generally small and non-significant. Notably, the largest group differences emerged for self-movement skills, suggesting that maturational transitions may particularly affect body management and balance-related abilities. An alteration in motor coordination around peak height velocity has been reported in the literature and is referred to as adolescent motor awkwardness [34]. Adolescent awkwardness is described as a transient and individual disruption of motor coordination around PHV, potentially linked to asynchronous growth and temporary neuromuscular reorganization, despite the lack of a unified definition in the literature [1]. Although these explanations are frequently cited in the literature, they do not fully clarify the nature or boundaries of the phenomenon [35]. Another interesting result of our study is that, overall, boys outperformed girls in the BMC. However, as shown in our previous analysis [25], even when compared by age, boys typically performed better in object-related tasks, while girls showed comparable or slightly better performance in self-movement in the Italian context. Another relevant finding of the present study is that, while both maturation and BMI z-score were independently associated with BMC, the strength or direction of this relationship was not altered by BMI z-score. These results suggest that the maturational influence on BMC is relatively consistent across the spectrum of ponderal status, with the observed curvilinear (U-shaped) trajectory remaining consistent irrespective of overweight children. To date, few studies have explicitly tested whether BMI z-score or other indicators of adiposity moderate the link between maturity and motor performance. Research has predominantly focused on main-effect associations, showing that overweight and obesity are negatively related to motor coordination and movement proficiency [10,36,37]. For example, Martins et al. [24] observed inverse associations between BMI z-score and MC in preschoolers but did not find evidence of differential patterns across maturational stages. Similarly, longitudinal analyses have demonstrated that overweight children exhibit consistently lower coordination levels over time, yet the maturational status of MC remains largely unchanged [37, 38]. Collectively, these findings and the current results indicate that, during middle childhood, body composition and biological maturation exert parallel rather than interactive effects on motor development.

Nonetheless, some limitations should be acknowledged. The crosssectional design precludes causal inference regarding the temporal sequence between maturation, overweight children, and MC. Longitudinal data would be required to confirm whether the observed U-shaped pattern persists across developmental transitions. The estimation of biological age using maturity offset equations, while practical and widely used in field settings, may introduce measurement error compared to radiographic or hormonal markers. Additionally, the sample, although large, was regionally confined to Northern Italy, and cultural or environmental factors influencing children’s movement opportunities may limit the extrapolation of results to other populations. Physical activity levels and training age beyond school physical education were not assessed and should be considered in future studies. Finally, while BMI z-score was included as a proxy for overweight children (adiposity), it does not distinguish between fat and lean mass; future research should incorporate more precise measures of body composition, such as skinfold thickness or bioimpedance analysis.

However, we should acknowledge several strengths of our study. First, it is among the few to investigate the non-linear association between biological maturation and BMC in a large sample of schoolaged children. The inclusion of both sexes and a broad age range, including pre- to post-PHV stages, enhances the generalizability of the findings across early and middle childhood. Moreover, the use of the MOBAK test battery allowed for an ecologically valid assessment of BMC directly relevant to the educational context, providing a clear picture of children’s functional MC. The analytical approach, incorporating quadratic regression and mediation modeling, further represents a methodological strength, as it captured non-linear developmental dynamics often overlooked by traditional linear models.

## CONCLUSIONS

The present study demonstrates a non-linear, U-shaped association between biological maturation and BMC, with on-time maturers showing lower BMC compared to their pre- and post-PHV peers. Moreover, while both maturation and BMI z-score were independently associated with MC, BMI did not mediate or moderate this relationship, suggesting that maturational effects on motor performance are largely independent of excess weight status. Further studies are needed to evaluate whether body fat, rather than ponderal status, could mediate this relationship. From a practical insight, these findings emphasize the importance of recognizing the PHV period as a sensitive developmental window. Tailored interventions focusing on coordination, balance, strength and movement variability during this phase may help counteract temporary declines in motor proficiency and support the consolidation of BMC. Such targeted approaches could promote long-term physical literacy and enhance sustained engagement in physical activity across childhood and adolescence.

## Supplementary Material

Early bloom, early boom? Motor competence profiles across maturation: a cross-sectional study
