# Supplementary material for: Early bloom, early boom? Motor competence profiles across maturation: a cross-sectional study
Source: Biol Sport. 2026 Jul 6;43:1547–57. doi: 10.5114/biolsport.2026.160855 (PMC13617485; doi:10.5114/biolsport.2026.160855)
Supplement: Supplementary file 1 [file JBS-43-57882-s1.pdf]

SUPPLEMENTARY MATERIALS

Table S1. Reporting checklist for cross sectional study.

		Reporting Item	Page Number
		Title and abstract	
Title	#1a	Indicate the study's design with a commonly used term in the title or the abstract	**1**
Abstract	#1b	Provide in the abstract an informative and balanced summary of what was done and what was found	**1–2**
		**Introduction**	
Background / rationale	#2	Explain the scientific background and rationale for the investigation being reported	**2**
Objectives	#3	State specific objectives, including any prespecified hypotheses	**3**
		**Methods**	
Study design	#4	Present key elements of study design early in the paper	**4**
Setting	#5	Describe the setting, locations, and relevant dates, including periods of recruitment, exposure, follow-up, and data collection	**5**
Eligibility criteria	#6a	Give the eligibility criteria, and the sources and methods of selection of participants.	**4**
	#7	Clearly define all outcomes, exposures, predictors, potential confounders, and effect modifiers. Give diagnostic criteria, if applicable	**4–5**
Data sources / measurement	#8	For each variable of interest give sources of data and details of methods of assessment (measurement). Describe comparability of assessment methods if there is more than one group. Give information separately for for exposed and unexposed groups if applicable.	**4–5**
Bias	#9	Describe any efforts to address potential sources of bias	**6**
Study size	#10	Explain how the study size was arrived at	**n/a**
Quantitative variables	#11	Explain how quantitative variables were handled in the analyses. If applicable, describe which groupings were chosen, and why	**6**
Statistical methods	#12a	Describe all statistical methods, including those used to control for confounding	**6**
Statistical methods	#12b	Describe any methods used to examine subgroups and interactions	**n/a**
Statistical methods	#12c	Explain how missing data were addressed	**4**
Statistical methods	#12d	If applicable, describe analytical methods taking account of sampling strategy	**n/a**
Statistical methods	#12e	Describe any sensitivity analyses	**6**
		**Results**	
Participants	#13a	Report numbers of individuals at each stage of study—eg numbers potentially eligible, examined for eligibility, confirmed eligible, included in the study, completing follow-up, and analysed. Give information separately for for exposed and unexposed groups if applicable.	**7**
Participants	#13b	Give reasons for non-participation at each stage	**n/a**
Participants	#13c	Consider use of a flow diagram	**n/a**
Descriptive data	#14a	Give characteristics of study participants (eg demographic, clinical, social) and information on exposures and potential confounders. Give information separately for exposed and unexposed groups if applicable.	**7**
Descriptive data	#14b	Indicate number of participants with missing data for each variable of interest	**4**
Outcome data	#15	Report numbers of outcome events or summary measures. Give information separately for exposed and unexposed groups if applicable.	**n/a**
Main results	#16a	Give unadjusted estimates and, if applicable, confounder-adjusted estimates and their precision (eg, 95% confidence interval). Make clear which confounders were adjusted for and why they were included	**7–8**
Main results	#16b	Report category boundaries when continuous variables were categorized	**n/a**
Main results	#16c	If relevant, consider translating estimates of relative risk into absolute risk for a meaningful time period	**n/a**
Other analyses	#17	Report other analyses done-e.g., analyses of subgroups and interactions, and sensitivity analyses	**n/a**

Table S1. Continue

		Reporting Item	Page Number
		**Discussion**	
Key results	#18	Summarise key results with reference to study objectives	**9**
Limitations	#19	Discuss limitations of the study, taking into account sources of potential bias or imprecision. Discuss both direction and magnitude of any potential bias.	**11**
Interpretation	#20	Give a cautious overall interpretation considering objectives, limitations, multiplicity of analyses, results from similar studies, and other relevant evidence.	**10–11**
Generalisability	#21	Discuss the generalisability (external validity) of the study results	**11**
		**Other Information**	
Funding	#22	Give the source of funding and the role of the funders for the present study and, if applicable, for the original study on which the present article is based	**n/a**

TABLE S2. Mediation Model Examining the Relationships Between Maturity Offset, BMI z-score, Sex, and Basic Motor Competencies.

1° Outcome	2° Outcome	Estimate (95% CI)	p-value
Maturity offset	BMI z-score	-0.040 (-0.078, 0.010)	0.153
Sex	BMI z-score	0.076 (0.026, 0.326)	**0.015**
BMI z-score	Basic motor competencies	-0.200 (-0.758, -0.410)	**< .001**
Maturity offset	Basic motor competencies	-0.240 (-0.752, -0.462)	**< .001**
Sex	Basic motor competencies	-0.038 (-0.685, 0.132)	0.200
Indirect effect	/	-0.040 (-0.078, 0.010)	0.166
Direct effect	/	0.076 (0.026, 0.326)	**< .001**
Total effect	/	-0.200 (-0.758, -0.410)	**< .001**

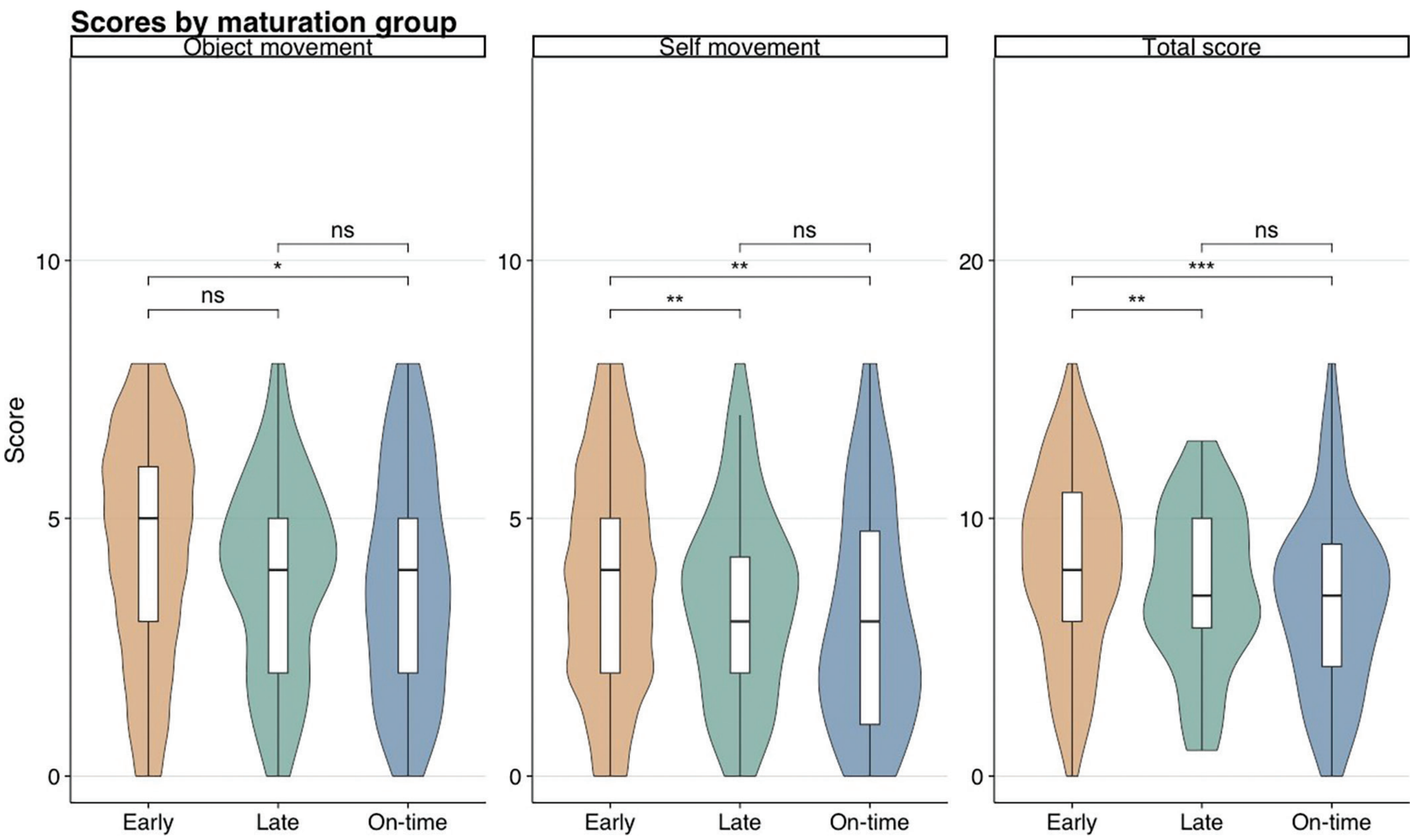

FIG. S1. Adjusted group differences in movement scores by maturation status.
Distributions of objective movement, self-reported movement and total scores are shown using violin plots with overlaid boxplots (median and interquartile range). Pairwise differences between maturation groups were evaluated using ANCOVA with sex as a covariate and Holm correction for multiple comparisons. Brackets indicate adjusted significance levels.

TABLE S3. Summary of MOBAK battery items.

Competence	Item	Section	Description
Object movement	throwing	MOBAK KG	*Throw a juggling balls six rounders from a scratch line marked 1.5 m in front of the target placed at 1.1 m height on a wall*
		MOBAK 1–2	*Throw juggling balls six rounders from a scratch line marked 2.0 m in front of the target placed at 1.3 m height on a wall*
		MOBAK 3–4	*Throw juggling balls six rounders from a scratch line marked 3.0 m in front of the target placed at 1.3 m height on a wall*
		MOBAK 5–6	*Throw juggling balls six rounders from a scratch line marked 3.5 m in front of the target placed at 1.3 m height on a wall*
	catching	MOBAK KG	*Catch the ball after the bounce about 1.1 m up on the floor before it falls to the ground*
		MOBAK 1–2	*Catch a rubber ball after a bounce on the ground.*
		MOBAK 3–4	*Throw a ball in the air behind the first line, follow the ball and catch it before it falls to the ground behind the second line.*
		MOBAK 5–6	*Throws a tennis ball at the wall and catch it directly when still in the air.*
	bouncing	MOBAK KG	*Bounce continuously the ball to the floor with both hands and catch it again without losing the ball*
		MOBAK 1–2	*Bounce a a basketball ball (N°3) through the corridor (5.0 m × 1.0m)*
		MOBAK 3–4	*Bounce a basketball ball (N°3) through the corridor (7.0 m × 1.4 m) with four obastacles.*
		MOBAK 5–6	*Bounce a basketball ball (N°6) quickly back and forth through an obstacle course with obstacle (8.0 m × 1.1 m)*
	dribbling	MOBAK KG	*Dribble a football ball along a corridor until the finish line without losing the ball*
		MOBAK 1–2	*Dribble a football ball (N°4) along a corridor (5.0 m × 1.0 m) until the finish line without losing the ball*
		MOBAK 3–4	*Dribble a football ball (N°4) along a corridor (7.5 m × 1.4 m) with obstacles until the turning point and then back again without losing the ball*
		MOBAK 5–6	*Dribble a football ball (N°4) quickly back and forth through an obstacle course (8.0 m × 1.1 m).*
Self-movement	balancing	MOBAK KG	*Balances forward over a reversed long bench from the first marking to the second marking, touch it and then balances backwards back to the first marking.*
		MOBAK 1–2	*Balance forwards and backwards across a see-sawing long bench*
		MOBAK 3–4	*Balance forwards and backwards over the long bench with 2 obstacles of 6 cm high*
		MOBAK 5–6	*Balance forwards and backwards over the long bench with 2 obstacles of 12 cm high*
	rolling	MOBAK KG	*From a squat down position, perform a roll forward on the downwardly inclined plane and come to a standstill on feet in one flow*
		MOBAK 1–2	From a squat position, perform *a roll forward*, with your hands placed on the mat.
		MOBAK 3–4	*Performs a roll forward over 2 longitudinal box pair*
		MOBAK 5–6	*From a two-legged stand position, perform a perform a diving forward roll over the Box and come to the starting position*
	Jumping	MOBAK KG	*From two-legged stand position behind the starting line continuously jump on one leg until the end line. Afterwards turn and jump on the other leg back to the starting line.*
		MOBAK 1–2	*Jump continuously across 4 tiles placed onthe floor, between the tiles standing on one foot while beneath the tiles jumping with straddled legs.*
		MOBAK 3–4	*Skip the rope without interruption for 20 seconds in place*
		MOBAK 5–6	*Skip the rope 20s with changing rhythms at 10s*
	Running	*MOBAK KG*	*Run forward in a corridor to the wall touching it and run backwards to the start line for two times*
		MOBAK 1–2	*Run fluently sideways from the first marker to the second on a 3.0 m line*
		MOBAK 3–4	*Run forward and sideways in a rectangular space (2.0 m × 4.0 m)*
		MOBAK 5–6	*Run forward and sideaway in a square space (4.0 m × 4.0 m) with three steps in each hoop placed on the floor when running forward*

Articles published in the Biology of Sport are licensed under an open access Creative Commons CC BY 4.0 license.
